# Supplementary material for: Genome‐wide off‐rates reveal how DNA binding dynamics shape transcription factor function
Source: Mol Syst Biol. 2020 Oct 14;16(10):e9885. doi: 10.15252/msb.20209885 (PMC7586999; doi:10.15252/msb.20209885)
Supplement: Supplementary file 1 — Expanded View Figures PDF [file MSB-16-e9885-s001.pdf]

Expanded View Figures

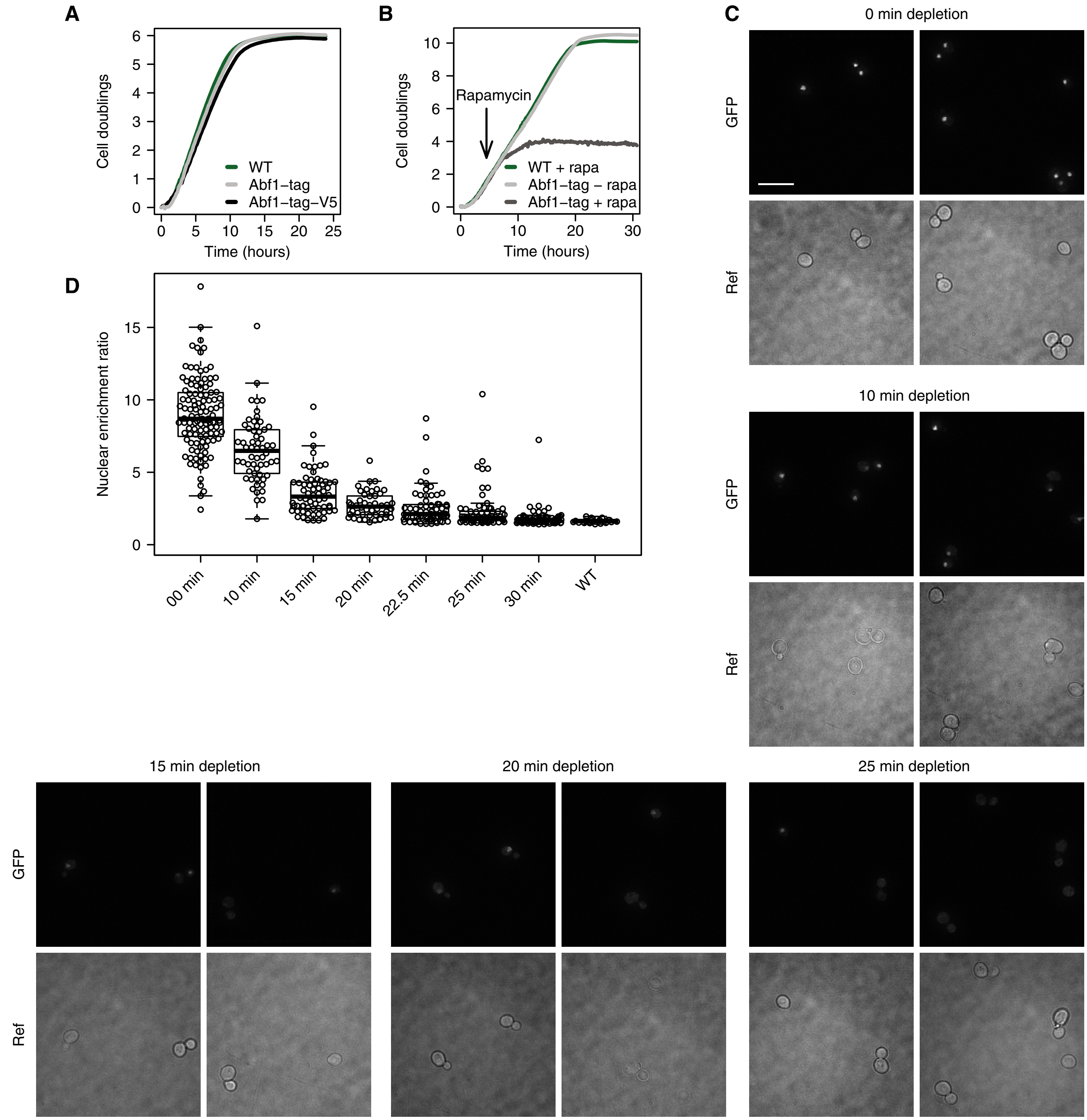

Figure EV1.

◀

Figure EV1. Dynamics of Abf1 depletion.

A Representative growth curves of the untagged parental background strain (WT), an *ABF1* FRB-GFP-tagged strain (Abf1-tag) and an *ABF1* FRB-GFP-V5-tagged strain (Abf1-tag-V5). The WT strain is the parental BY4742/S288C strain of the Abf1 anchor-away strains that have been genetically desensitized to rapamycin. The Abf1-tag strain grows approximately 10% slower compared to the WT strain and the Abf1-tag-V5 strain grows approximately 15% slower than the WT strain.

B Growth curves of the WT strain with rapamycin, and the *ABF1* FRB-GFP tagged strain with and without rapamycin, the inducing agent for nuclear depletion (Haruki *et al*, 2008). Rapamycin was added 4.5 h after the start of the experiment to induce nuclear depletion (arrow). Upon induction of depletion, clear disruption of growth is visible after approximately 3 h (t = 7.5 h) and cessation of growth is visible after approximately 8 h (t = 12.5 h).

C Representative fluorescence microscopy images of *ABF1* FRB-GFP-V5 cells before (0 min) and at several time points after (10, 15, 20 and 25 min) induction of nuclear depletion. Scale bar: 10 μm.

D Quantification of nuclear enrichment ratios of *ABF1* FRB-GFP and *ABF1* FRB-GFP-V5 cells during depletion (three biological replicates). Both DNA-bound and free Abf1 contribute to the GFP signal and therefore the decrease in fluorescence represents the depletion speed of both unbound as well as bound Abf1. Dots represent individual cell measurements. The nuclear enrichment ratio was calculated as the maximum intensity divided by the median intensity of each cell. A WT strain without GFP was taken along as a control.

© 2020 The Authors

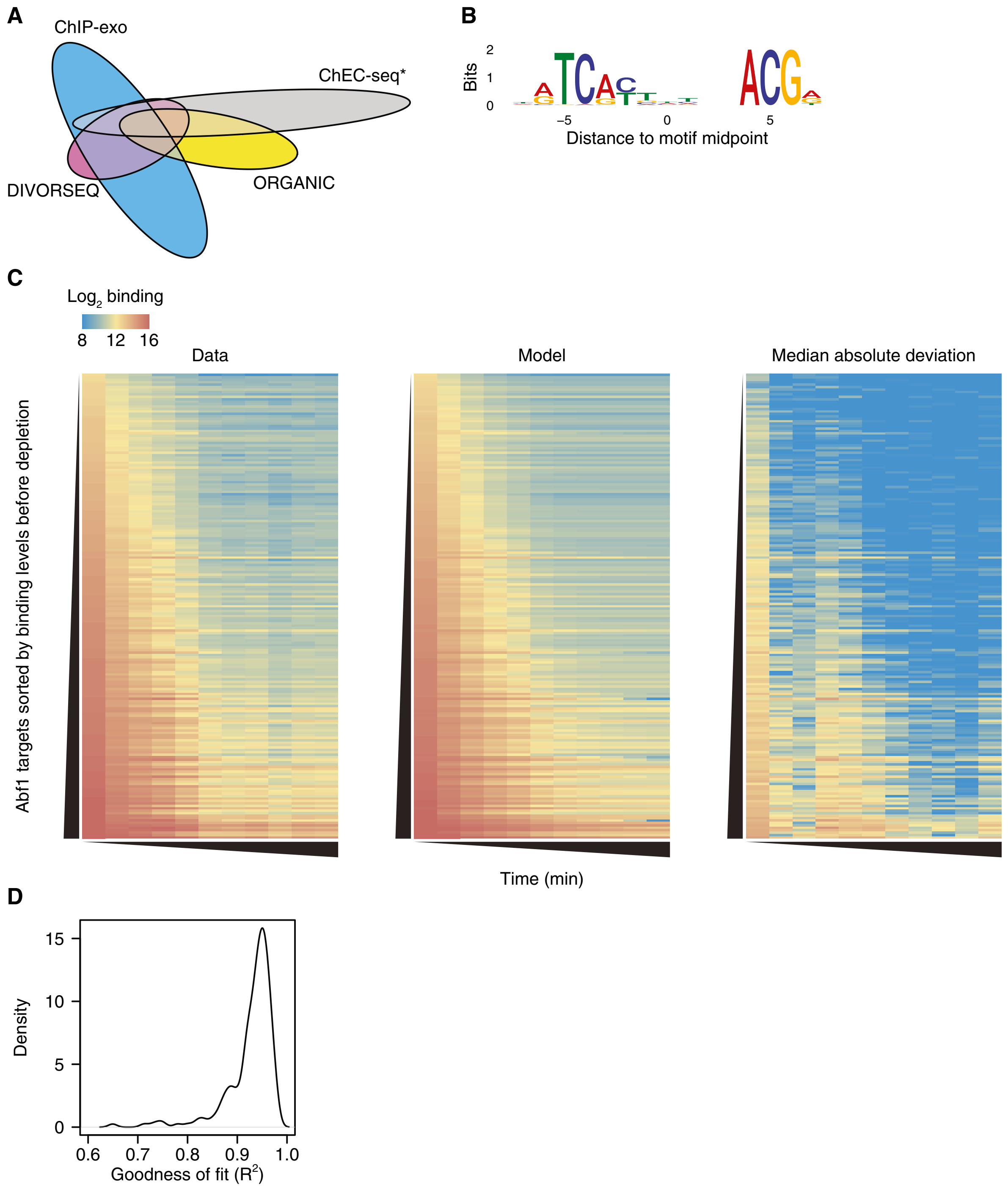

Figure EV2. The exponential decay model closely fits the binding data.

A Venn diagram showing the overlap between unfiltered Abf1 binding sites detected here (n = 948) and published Abf1 binding sites detected using ORGANIC ChIP (Kasinathan *et al*, 2014; n = 1,068), binding sites detected using ChEC-seq that were annotated both as "fast" and "high scoring" (Zentner *et al*, 2015; n = 1,583) and binding sites that were detected using ChIP-exo v5 (Rossi *et al*, 2018b; n = 3,177).

B Consensus motif of all Abf1 motifs found at the 191 binding sites. The orientation is the same as the motif used to search for matches (MacIsaac *et al*, 2006), which is the reverse complement of the motif used in (Rossi *et al*, 2018a).

C Heatmap representation of the average Abf1 binding at the 191 binding sites, quantified as the $\log_2$ number of reads in the peak (peak summit ± 50 bp). The rows are sorted on the binding levels before depletion, and the columns represent increasing time of depletion. From left to right: average binding data, fit and residuals of the fit (median absolute deviation) are shown.

D The distribution of the goodness of fit of the 191 Abf1 binding sites.

Figure EV3. Changes in mRNA synthesis correspond more closely to off-rates than binding levels.

A Nucleosome occupancy of example genes relative to the +1 nucleosome dyad. Genes are shown from each of the residence time quartiles (rows), with different expression changes (columns), with strong changes (fold change > 2 at t = 20, left), weak changes (1.5 ≤ fold change ≤ 2 at t = 20, middle) or no changes (fold change < 1.5 at t = 20, right). Nucleosome occupancy is shown before (light grey fill) and after (dashed line) Abf1 depletion. Nucleosome binding data and +1 nucleosome positions are from (Kubik *et al*, 2015).

B Change in mRNA synthesis rate versus Abf1 binding level before depletion of the corresponding binding site after 5, 10, 15, 20 and 30 min of depletion of downregulated genes (fold change > 1.5 and P < 0.01 at 20 and 30 min of depletion) with Abf1 binding in the promoter (n = 88). The panel for t = 10 min is also shown in Fig 3E.

C Change in mRNA synthesis rate versus the off-rates of the corresponding binding sites for the same gene-binding sites pairs as in (A) 5, 10, 15, 20 and 30 min after induction of depletion. The panel for t = 10 min is also shown in Fig 3F.

© 2020 The Authors

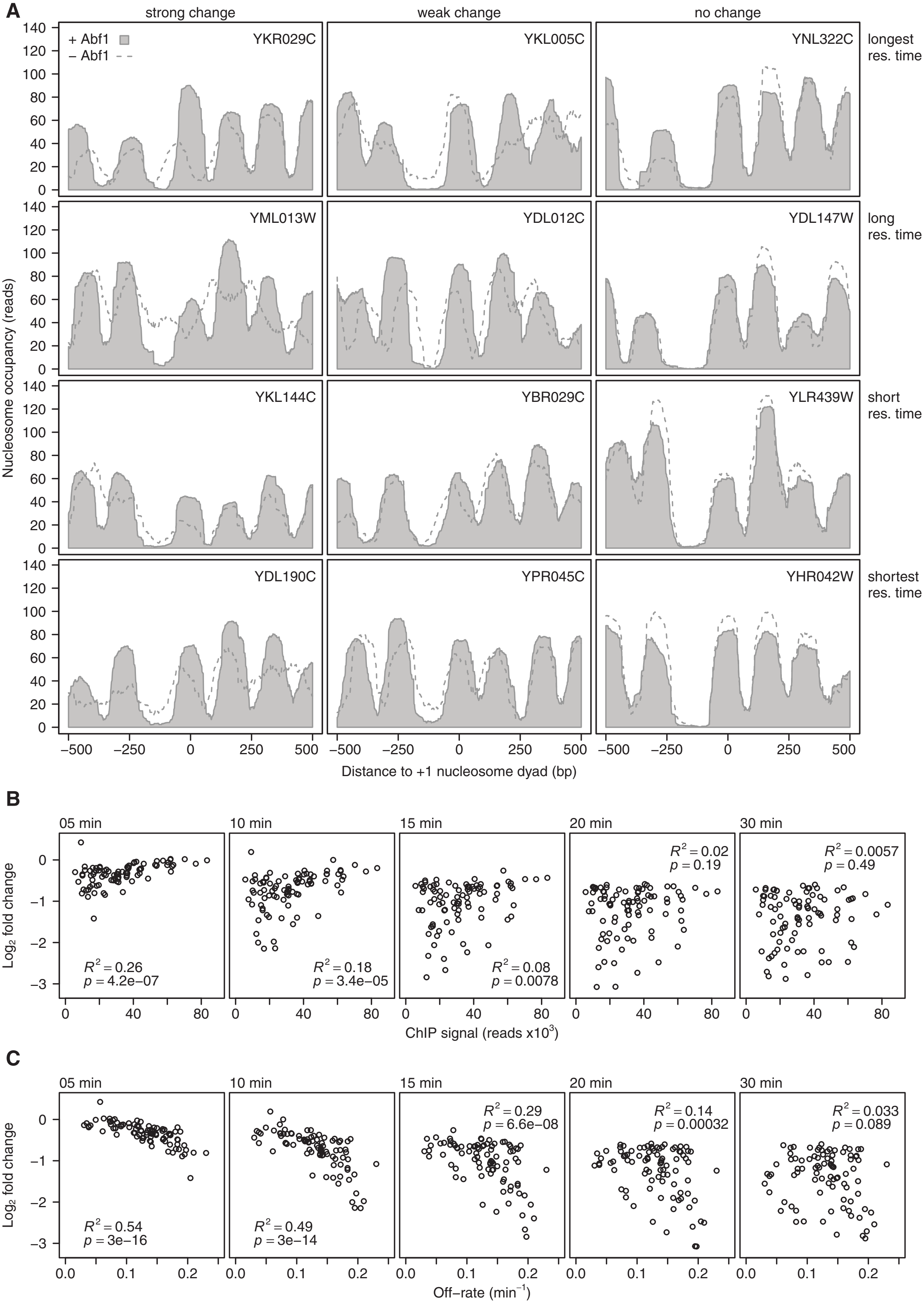
A
strong change
weak change
no change
+ Abf1
− Abf1
YKR029C
YKL005C
YNL322C
longest res. time
YML013W
YDL012C
YDL147W
long res. time
YKL144C
YBR029C
YLR439W
short res. time
YDL190C
YPR045C
YHR042W
shortest res. time
Nucleosome occupancy (reads)
Distance to +1 nucleosome dyad (bp)
B
05 min
10 min
15 min
20 min
30 min
Log2 fold change
R2 = 0.26
p = 4.2e−07
R2 = 0.18
p = 3.4e−05
R2 = 0.08
p = 0.0078
R2 = 0.02
p = 0.19
R2 = 0.0057
p = 0.49
ChIP signal (reads x103)
C
R2 = 0.54
p = 3e−16
R2 = 0.49
p = 3e−14
R2 = 0.29
p = 6.6e−08
R2 = 0.14
p = 0.00032
R2 = 0.033
p = 0.089
Off−rate (min−1)

Figure EV3.
